# *Xylaria iriomotensis* sp. nov. from termite nests and notes on *X. angulosa*

**DOI:** 10.1186/s40529-024-00447-7

**Published:** 2025-01-17

**Authors:** Izumi Okane, Huei-Mei Hsieh, Yu-Ming Ju, Chun-Ru Lin, Chun-Yun Huang, I-Ching Kuan

**Affiliations:** 1 Institute of Life and Environmental Sciences, University of Tsukuba, Tsukuba, 305- 8577 Ibaraki Japan; 2 Institute of Plant and Microbial Biology, Academia Sinica, Nankang, 11529 Taipei Taiwan; 3 Department of Chemical Engineering and Biotechnology, Tatung University, Taipei, 10491 Taiwan

**Keywords:** *Odontotermes formosanus*, Subgenus *Pseudoxylaria*, Systematics, Xylariaceae

## Abstract

**Background:**

Fungus gardens of the termite *Odontotermes formosanus*, excavated from Iriomote Island, Okinawa Prefecture, Japan, were subsequently incubated under laboratory conditions. A *Xylaria* species emerging from these fungus gardens was initially identified as *X. angulosa*, a species originally described from North Sulawesi, Indonesia. The Iriomote fungus is now described as a distinct species, *X. iriomotensis*.

**Results:**

*Xylaria iriomotensis* is peculiar in producing the teleomorph in culture but lacking an anamorph. Cultures of *X. angulosa* were obtained from two Taiwan specimens, which agree with the holotype from BO and the isotypes from NY and WSP in their stromata being repeatedly dichotomously branched and possessing a black core. In contrast to *X. iriomotensis*, *X. angulosa* does not form the teleomorph in culture but a typical *Xylaria* anamorph with conidiophores densely arranged in palisades. The ITS sequence obtained from the WSP isotype shared high similarities with those two Taiwan specimens as well as an Indian specimen, reconfirming the latter three specimens as *X. angulosa*. These four specimens shared 98.28–99.66% similarities at ITS sequences among themselves but only 84.25–85.01% similarities with *X. iriomotensis*. Molecular phylogenetic studies based on sequences of multiple protein-coding loci indicate that, while *X. iriomotensis* is grouped with three soil-dwelling species of the *X. guepini* cluster, *X. angulosa* belongs to the *X. nigripes* cluster, which includes all known species capable of producing massive sclerotia.

**Conclusion:**

*Xylaria iriomotensis* has the teleomorph known only in culture, remaining to be rediscovered in its natural habitat where the stromatal morphology may be somewhat varied. The geographic distribution of *X. angulosa*, previously known only in Indonesia, has been expanded to Taiwan and India. *Xylaria angulosa* grouping with the *X. nigripes* cluster in our phylogenetic analyses indicates its potential to form massive sclerotia within termite nests.

## Background

Xylariaceae Tul. & C. Tul. (Xylariales, Sordariomycetes, Ascomycota) contains more than a thousand of described species that are characterized by mid- to large-sized stromata and display diversified life styles (Rogers 1979, 2000; Suwannasai et al. 2023; Whalley 1985, 1996). *Xylaria* Hill ex Schrank, commonly found in the tropics and subtropics (Ju and Rogers 1999; Rajtar et al. 2023; Rogers et al. 1987, 1988; San Martín and Rogers 1989; Van der Gucht 1995; Vandegrift et al. 2023), is a highly diversified genus in the Xylariaceae, where species are characterized by dark, one-celled ascospores with a germ slit and unitunicate asci topped with a ring-like apparatus staining blue in an iodine reagent. Most *Xylaria* species are found on dead wood (Fournier et al. 2018, 2019, 2020), and fewer species grow on fallen fruits and seeds (Ju et al. 2018), dead leaves and petioles (Ju and Hsieh 2023), and termite nests and soil (Hsieh et al. 2024; Ju and Hsieh 2007; Ju et al. 2022, 2023; Rogers et al. 2005; Wangsawat et al. 2021).

*Xylaria* species inhabiting termite nests and soil belong to the subgenus *Pseudoxylaria*, where approximately 40 species are included. Most of the members are associated with termite nests (Ju and Hsieh 2007; Ju et al. 2022, 2023; Rogers et al. 2005; Wangsawat et al. 2021) and only four are associated with soil (Chou et al. 2017; Hsieh et al. 2022; Ju et al. 2011; Kim et al. 2016). While the stromata of these species display a wide range of variation: delicate to stout, with an acute to rounded apex, stipitate to sessile, unbranched to branched, smooth to roughened, their ascospores are quite consistent in being small, shorter than 8 μm in most cases, and their ostioles are coarsely conic-papillate.

Okane and Nakagiri (2007) reported two xylariaceous fungi from termite nests of *Odontotermes formosanus* in Iriomote Island, Okinawa Pref., Japan: *Geniculisynnema termiticola* Okane & Nakagiri and *X. angulosa* J. D. Rogers, Callan & Samuels. These two species produced stromata from the excavated nests, which were brought back to the laboratory and incubated in moist chambers. *Geniculisynnema termiticola* is an anamorphic *Xylaria* species and was recombined with the genus *Xylaria* to form *X*. *termiticola* (Okane & Nakagiri) Y.-M. Ju (Réblová et al. 2016) to comply with the change to the International Code of Nomenclature for algae, fungi, and plants (McNeill et al. 2012), where two or more names for different morphs of the same taxon are no longer allowed. The material identified as *X*. *angulosa* was based on the teleomorph produced on agar media, where an anamorph is lacking.

In this study, we compared the Iriomote material with the type material of *X. angulosa* from North Sulawesi, Indonesia (Rogers et al. 1987) and found it distinctive from the latter. As the Iriomote material does not fit a known species, we thus describe it as a new species, *X. iriomotensis*. We also provide teleomorphic, anamorphic, and culture descriptions of *X*. *angulosa* and inferred its phylogenetic relationships with *X*. *iriomotensis* in the context of numerous other *Xylaria* species using sequences of three protein-coding loci.

## Methods

### Fungal material and observation

While stromata of *X*. *angulosa* came from field-collected as well as loaned herbarium specimens, those of *X. iriomotensis* were formed under laboratory conditions by incubating fungus gardens of *Odontotermes formosanus* in a moist chamber. The fungus gardens, from which *X*. *iriomotensis* emerged, were collected from Iriomote Island, Okinawa Pref., Japan, as documented in Okane and Nakagiri (2007). The brown stromata of *X. iriomotensis* were formed among other whitish synnemata within a week. To obtain cultures of *X*. *iriomotensis* and *X*. *angulosa*, the stromatal tissue of these two species was placed on oatmeal agar (OMA), potato dextrose agar (PDA), and 2% malt extract agar (MEA) without adding peptone (Kenerley and Rogers 1976). Resulting colonies were transferred to 9-cm plastic Petri dishes containing 2% OMA for culture descriptions and incubated at 20 °C under 12 h fluorescent light. Asci, ascospores, conidiogenous cells, and conidia were examined using differential interference contrast microscopy (DIC) and bright field microscopy (BF). Material was mounted in water and Melzer’s iodine reagent for examination by DIC and BF. The cultures of *X*. *angulosa* and *X. iriomotensis* are available at Bioresource Collection and Research Center in Taiwan (BCRC) and Biological Resource Center of National Institute of Technology and Evaluation in Japan (NBRC), respectively. The holotype of *X*. *iriomotensis* was harvested from the stromata produced on OMA.

### Analyzing ITS sequences

Sequences of nuclear rDNA internal transcribed spacers (ITS = ITS1-5.8 S-ITS2) were obtained according to Hsieh et al. (2009). To reconfirm that *X*. *iriomotensis* is not conspecific with *X*. *angulosa*, we compared its ITS sequence with that from the isotype of *X. angulosa* deposited at WSP. We then analyzed these sequences with the ITS sequences of various species from termite nests and soil that we had obtained (the italicized ITS sequences listed in Table 1) using Maximum-Likelihood (ML) and Bayesian Inference (BI) phylogenetic methods, with ML and BI trees generated using RAxML analysis ver. 8.2.10 (Stamatakis 2014) and MrBayes ver. 3.2.6 (Ronquist et al. 2012), respectively, as detailed in Ju et al. (2023). The outgroup was *X*. *hypoxylon* (L.) Grev.

**Table 1:** Sequences of taxa and isolates included in the ML and BI analyses

Taxon	Origin	Collecting data	GenBank accession number			
			β-tubulin gene	α-actin gene	RPB2 gene	ITS
*Amphirosellinia fushanensis* Y.-M. Ju et al.	Taiwan	HOLOTYPE (Ju et al. 2004)	GQ495950	GQ452360	GQ848339	GU339496
*Am. nigrospora* Y.-M. Ju et al.	Taiwan	HOLOTYPE (Ju et al. 2004)	GQ495951	GQ452361	GQ848340	GU322457
*Astrocystis bambusae* (Henn.) Læssøe & Spooner	Taiwan	*Ju & Hsieh 89021904* (Hsieh et al. 2010)	GQ495942	GQ449239	GQ844836	GU322449
*As. mirabilis* Berk. & Broome	Taiwan	*Ju & Hsieh 94070803* (Hsieh et al. 2010)	GQ495941	GQ449238	GQ844835	GU322448
*As. sublimbata* (Durieu & Mont.) G. C. Hughes	Taiwan	*Ju & Hsieh 89032207* (Hsieh et al. 2010)	GQ495940	GQ449236	GQ844834	GU322447
*Biscogniauxia arima* San Martín et al.	Mexico	*YMJ122* from ISOTYPE (Hsieh et al. 2005; Ju et al. 1998)	AY951672	AY951784	GQ304736	EF026150
*Discoxylaria myrmecophila* J. C. Lindq. & J. E. Wright	Mexico	*YMJ169* from *Moreno 713* (Rogers et al. 1995)	GQ487710	GQ438747	GQ844819	GU322433
*Entoleuca mammata* (Wahlenb.) J. D. Rogers & Y.-M. Ju	France	*YMJ100* from *Candoussau*,* F. 5254* (Hsieh et al. 2010)	GQ470230	GQ398230	GQ844782	GU300072
*Euepixylon sphaeriostomum* (Schwein.) Y.-M. Ju & J. D. Rogers	USA	*YMJ261* from *Huhndorf*,* S. M. 1447* (Hsieh et al. 2010)	GQ470224	GQ389696	GQ844774	GU292821
*Kretzschmaria clavus* (Fr.) Sacc.	French Guiana	*YMJ114* from *Huhndorf 803* (Ju et al. 2007; Rogers and Ju 1998)	EF025611	EF025596	GQ844789	EF026126
*K. guyanensis* J. D. Rogers & Y.-M. Ju	Taiwan	*Ju & Hsieh 89062903* (Hsieh et al. 2010)	GQ478214	GQ408901	GQ844792	GU300079
*K. lucidula* (Mont.) Dennis	French Guiana	*YMJ112* from *Huhndorf 677* (Ju et al. 2007; Rogers and Ju 1998)	EF025610	EF025595	GQ844790	EF026125
*K. megalospora* J. D. Rogers & Y.-M. Ju	Malaysia	*YMJ229* from *Whalley*,* M. FH 64−97* (Ju et al. 2007)	EF025609	EF025594	GQ844791	EF026124
*K. neocaledonica* (Har. & Pat.) J. D. Rogers & Y.-M. Ju	Taiwan	*Guu*,* J.-R. 94031003* (Hsieh et al. 2010)	GQ478213	GQ398236	GQ844788	GU300078
*K. pavimentosa* (Ces.) P. Martin	Taiwan	*YMJ109* from *Wang 511* (Rogers and Ju 1998)	GQ478212	GQ398235	GQ844787	GU300077
*K. sandvicensis* (Reichardt) J. D. Rogers & Y.-M. Ju	USA, Hawaiian Islands	*YMJ113* from *Rogers/4 Jan 1996* (Rogers and Ju 1998)	GQ478211	GQ398234	GQ844786	GU300076
*Nemania abortiva* J. D. Rogers et al.	USA, Hawaiian Islands	*YMJ467* from HOLOTYPE (Rogers et al. 2005)	GQ470219	GQ374123	GQ844768	GU292816
*N. beaumontii* (Berk. & M. A. Curtis) Y.-M. Ju & J. D. Rogers	French West Indies	*YMJ405* from *Lechat*,* C. CLL2039* (Fournier et al. 2018; Hsieh et al. 2010)	GQ470222	GQ389694	GQ844772	GU292819
*N. bipapillata* (Berk. & M. A. Curtis) Pouzar	Taiwan	*Ju & Hsieh 90080610* (Hsieh et al. 2010)	GQ470221	GQ389693	GQ844771	GU292818
*N. diffusa* (Sowerby) S. F. Gray	Taiwan	*Ju & Hsieh 91020401* (Hsieh et al. 2010)	GQ470220	GQ389692	GQ844769	GU292817
*N. illita* (Schwein.) Pouzar	USA	*YMJ236* from *Tsai*,* S.-J.* (Ju et al. 2007)	EF025608	EF025593	GQ844770	EF026122
*N. macrocarpa* Y.-M. Ju & J. D. Rogers	USA, Hawaiian Islands	*YMJ265* from HOLOTYPE (Ju and Rogers 2002)	GQ470226	GQ389698	GQ844776	GU292823
*N. maritima* Y.-M. Ju & J. D. Rogers	Taiwan	HOLOTYPE (Ju and Rogers 2002)	GQ470225	GQ389697	GQ844775	GU292822
*N. primolutea* Y.-M. Ju et al.	Taiwan	HOLOTYPE (Ju et al. 2005; Ju et al. 2007)	EF025607	EF025592	GQ844767	EF026121
*N. serpens* (Pers.) S. F. Gray (Barron isolate)	Canada	*YMJ235* from *Barron*,* G.*, as *Hypoxylon* in Petrini and Rogers (1986)	GQ470223	GQ389695	GQ844773	GU292820
*Rosellinia buxi* Fabre	France	*YMJ99* from *Candoussau*,* F.* (Hsieh et al. 2010)	GQ470228	GQ398228	GQ844780	GU300070
*R. lamprostoma* Syd. & P. Syd.	Taiwan	*Ju & Hsieh 89112602* (Ju et al. 2007)	EF025604	EF025589	GQ844778	EF026118
*R. merrillii* Syd. & P. Syd.	Taiwan	*Ju & Hsieh 89112601* (Hsieh et al. 2010)	GQ470229	GQ398229	GQ844781	GU300071
*R. necatrix* (R. Hartig) Berl.	Taiwan	*Ju & Hsieh 89062904* (Ju et al. 2007)	EF025603	EF025588	GQ844779	EF026117
*R. sanctaecruciana* Ferd. & Winge	Taiwan	*Ju & Hsieh 90072903* (Hsieh et al. 2010)	GQ470227	GQ389699	GQ844777	GU292824
*Stilbohypoxylon elaeidicola* (Henn.) L. E. Petrini	French Guiana	*YMJ173* from *Huhndorf 928* (Ju et al. 2007), as *S. moelleri* in Rogers and Ju (1997)	EF025616	EF025601	GQ844826	EF026148
*S. elaeidicola* (Henn.) L. E. Petrini	Taiwan	*Ju & Hsieh 94082615* (Hsieh et al. 2010)	GQ495933	GQ438754	GQ844827	GU322440
*S. quisquiliarum* (Mont.) J. D. Rogers & Y.-M. Ju	French Guiana	*YMJ172* from *Huhndorf 940* (Ju et al. 2007; Rogers and Ju 1997)	EF025605	EF025590	GQ853020	EF026119
*S. quisquiliarum* (Mont.) J. D. Rogers & Y.-M. Ju	Taiwan	*Ju & Hsieh 89091608* (Ju et al. 2007)	EF025606	EF025591	GQ853021	EF026120
*Xylaria acuminatilongissima* Y.-M. Ju & H.-M. Hsieh	Taiwan	*YMJ623* from HOLOTYPE (Ju and Hsieh 2007)	GQ502711	GQ853046	GQ853028	*EU178738*
*X. adscendens* (Fr.) Fr.	French West Indies	*YMJ570* from *Lechat*,* C. CLL5347* (Hsieh et al. 2010)	GQ487708	GQ438745	GQ844817	GU300101
*X. adscendens* (Fr.) Fr.	Thailand	*YMJ865* from *Bandoni*,* R. J.*,* Bandoni*,* A. A. & Flegel*,* T. W. 12017* (Hsieh et al. 2010)	GQ487709	GQ438746	GQ844818	GU322432
*X. alboareolata Y.-M. Ju & J.D. Rogers*	French West Indies	*YMJ543* from *Chabrol*,* J. CLL5372* (Fournier et al. 2018b), *as X. areolata* in Hsieh et al. (2010)	GQ478215	GQ408902	GQ844793	GU300080
*X. allantoidea* (Berk.) Fr.	Taiwan	*Ju & Hsieh 94042903* (Hsieh et al. 2010)	GQ502692	GQ452377	GQ848356	GU324743
*X. amphithele* San Martín & J. D. Rogers	French West Indies	*YMJ529* from *Lechat*,* C. CLL5352* (Hsieh et al. 2010)	GQ478218	GQ408905	GQ844796	GU300083
*X. angulosa* J. D. Rogers et al.	Indonesia	ISOTYPE (WSP) (Rogers et al. 1987)	–	**PQ605199**	–	***PQ582215***
*X. angulosa* J. D. Rogers et al.	India	*YMJ733* (the present study)	–	**PQ605198**	–	***PQ582214***
*X. angulosa* J. D. Rogers et al.		*YMJ1199* (the present study)	**PQ605183**	**PQ605193**	**PQ605188**	***PQ582212***
*X. angulosa* J. D. Rogers et al.		*YMJ3267* (the present study)	**PQ605184**	**PQ605194**	**PQ605189**	***PQ582213***
*X. apoda* (Berk. & Broome) J. D. Rogers & Y.-M. Ju	Taiwan	*Ju & Hsieh 90080804* (Hsieh et al. 2010)	GQ495930	GQ438751	GQ844823	GU322437
*X. arbuscula* Sacc.	Taiwan	*Ju & Hsieh 89041211* (Hsieh et al. 2010)	GQ478226	GQ421286	GQ844805	GU300090
*X. arbuscula* var. *plenofissura* Y.-M. Ju & S.-S. Tzean	Taiwan	*Ju & Hsieh 93082814* (Hsieh et al. 2010)	GQ478225	GQ421285	GQ844804	GU339495
*X. aristata* Mont. var. *aristata*	Taiwan	*Ju & Hsieh 90071613*, as *X. sicula* f. *major* in Hsieh et al. (2010)	GQ478216	GQ408903	GQ844794	GU300081
*X. atrodivaricata* Y.-M. Ju & H.-M. Hsieh	Taiwan	*YMJ615* from HOLOTYPE (Ju and Hsieh 2007)	GQ502713	GQ853048	GQ853030	*EU178739*
*X. atrosphaerica* (Cooke & Massee) Callan & J. D. Rogers	Taiwan	*Ju & Hsieh 91111214* (Hsieh et al. 2010)	GQ495953	GQ452363	GQ848342	GU322459
*X. badia* Pat.	Taiwan	*Ju & Hsieh 95070101* (Hsieh et al. 2010)	GQ495939	GQ449235	GQ844833	GU322446
*X. bambusicola* Y.-M. Ju & J. D. Rogers	Taiwan	*YMJ205* from HOLOTYPE (Hsieh et al. 2005; Ju and Rogers 1999)	AY951762	AY951873	GQ844802	EF026123
*X. bambusicola* Y.-M. Ju & J. D. Rogers	Thailand	*YMJ162* from *Bandoni*,* R. J. & A. A. et al.* (Hsieh et al. 2010)	GQ478223	GQ408910	GQ844801	GU300088
*X. berteri* (Mont.) Cooke	USA, Hawaiian Islands	*YMJ256* from *Rogers*,* J. D. K-1* (Hsieh et al. 2010)	GQ502698	GQ455442	GQ848363	GU324750
*X. berteri* (Mont.) Cooke	Taiwan	*Ju & Hsieh 90112623* (Hsieh et al. 2005)	AY951763	AY951874	GQ848362	GU324749
*X. brevifurcata* Y.-M. Ju & H.-M. Hsieh	Taiwan	*YMJ986* (Ju et al. 2023)	OQ818431	OQ818371	OQ851525	*OQ845481*
*X. brevifurcata* Y.-M. Ju & H.-M. Hsieh	Taiwan	*YMJ1381* from HOLOTYPE (Ju et al. 2023)	OQ818432	OQ818372	OQ851526	*OQ845482*
*X. brunneovinosa* Y.-M. Ju & H.-M. Hsieh	Taiwan	*YMJ720* from HOLOTYPE (Ju and Hsieh 2007)	GQ502706	GQ853041	GQ853023	*EU179862*
*X. cantareirensis* (Henn.) J. Fourn. & Lechat	French West Indies	*YMJ526* from *Lechat*,* C. CLL5437* (Fournier et al. 2018b), as *Penzigia* in Hsieh et al. (2010)	GQ478220	GQ408907	GQ844798	GU300085
*X. castorea* Berk.	New Zealand	*YMJ600* from *Samuels 85−75* (Hsieh et al. 2010)	GQ502703	GQ455447	GQ853018	GU324751
*X.* cf. *castorea* Berk.	Taiwan	*Ju & Hsieh 91092303* (Hsieh et al. 2010)	GQ502704	GQ455448	GQ853019	GU324752
*X. chaiyaphumensis* Wangsawat et al.	Thailand	*SWUF16-11.4* (Wangsawat et al. 2021)	OQ845433	OQ845425	OQ851585	*MT622776*
*X. chaiyaphumensis* Wangsawat et al.	Thailand	*SWUF17-49.2* from HOLOTYPE (Wangsawat et al. 2021)	OQ845434	OQ845426	OQ851586	*MT622775*
*X. cirrata* Pat.	Taiwan	*YMJ664* from EPITYPE (Ju and Hsieh 2007)	GQ502707	GQ853042	GQ853024	*EU179863*
*X. coccophora* Mont.	French Guiana	*YMJ786* from *Lechat*,* C. CLL7056* (Hsieh et al. 2010)	GQ487701	GQ421289	GQ844809	GU300093
*X. conica* N. Wangsawat et al.	Thailand	HOLOTYPE (Wangsawat et al. 2021)	–	–	–	*MT622787*
*X. coprinicola* Y.-M. Ju et al.	China	*YMJ1145* from HOLOTYPE (Ju et al. 2011)	HM585018	HM585017	HM585019	*HM585020*
*X. cranioides* (Sacc. & Paol.) Dennis	Taiwan	*YMJ226* from *Wen 712* (Ju and Rogers 2001)	GQ478210	GQ398233	GQ844785	GU300075
*X. crozonensis* P. Leroy & Mornand	France	*YMJ398* from *Mornand*,* F. JF04151* (Hsieh et al. 2010)	GQ502697	GQ455441	GQ848361	GU324748
*X. cubensis* (Mont.) Fr.	French West Indies	*YMJ419* from *Lechat*,* C. CLL2179* (Fournier et al. 2019), as *X*. *laevis* in Hsieh et al. (2010)	GQ502695	GQ455439	GQ848359	GU324746
*X. cubensis* (Mont.) Fr.	Taiwan	*Ju & Hsieh 95072910*, as *X*. *laevis* in Hsieh et al. (2010)	GQ502696	GQ455440	GQ848360	GU324747
*X. culleniae* Berk. & Broome	Thailand	*YMJ189* from *Whalley*,* M. F.NH9* (Hsieh et al. 2010)	GQ495935	GQ438756	GQ844829	GU322442
*X. cuneata* C. G. Lloyd	French West Indies	*YMJ495* from *Lechat*,* C. CLL5131* (Fournier et al. 2019), as *X*. *montagnei* Hamme & Guerrero in Hsieh et al. (2010)	GQ495948	GQ449244	GQ848337	GU322455
*X. curta* Fr.	French West Indies	*YMJ494* from *Lechat*,* C. CLL5044* (Hsieh et al. 2010)	GQ495937	GQ449233	GQ844831	GU322444
*X. curta* Fr.	Taiwan	*Ju & Hsieh 92092022* (Hsieh et al. 2010)	GQ495936	GQ438757	GQ844830	GU322443
*X. digitata* (L.) Grev.	Ukraine	*YMJ919* from *Prilutsky*,* O.* (Hsieh et al. 2010)	GQ495949	GQ449245	GQ848338	GU322456
*X. enterogena* (Mont.) Fr.	French Guiana	*YMJ785* from *Lechat*,* C. CLL7043* (Hsieh et al. 2010)	GQ502685	GQ452370	GQ848349	GU324736
*X. escharoidea* (Berk.) Fr.	Taiwan	*YMJ658* from EPITYPE (Ju and Hsieh 2007)	GQ502709	GQ853044	GQ853026	*EU179864*
*X. feejeensis* (Berk.) Fr.	French West Indies	*YMJ565* from *Lechat*,* C. CLL5653* (Hsieh et al. 2010)	GQ495945	GQ449241	GQ848334	GU322452
*X. feejeensis* (Berk.) Fr.	Taiwan	*Ju & Hsieh 92092013* (Hsieh et al. 2010)	GQ495947	GQ449243	GQ848336	GU322454
*X. feejeensis* (Berk.) Fr.	Thailand	*YMJ180* from *Whalley*,* M. UN515* (Hsieh et al. 2010)	GQ495946	GQ449242	GQ848335	GU322453
*X. fimbriata* C. G. Lloyd	French West Indies	*YMJ491* from *Lechat*,* C. CLL5010* (Hsieh et al. 2010)	GQ502705	GQ853040	GQ853022	*GU324753*
*X. fissilis* Ces.	French West Indies	*YMJ367* from *Lechat*,* C. CLL0928* (Hsieh et al. 2010)	GQ470231	GQ398231	GQ844783	GU300073
*X. flabelliformis* (Schwein.) Fr.	USA	*YMJ860* from *Rogers*,* J. D.*, as *X*. *cubensis* in Hsieh et al. (2010)	GQ502700	GQ455444	GQ848365	GU991523
*X. flabelliformis* (Schwein.) Fr.	Papua New Guinea	*YMJ159* from *Van der Gucht & De Meester 92−521*, as *X*. *cubensis* in Van der Gucht (1995) and Hsieh et al. (2010)	GQ502702	GQ455446	GQ853017	MZ854247
*X. flabelliformis* (Schwein.) Fr.	Russian Far East	*YMJ477* from *Vasilyeva*,* L. N.*, as *X*. *cubensis* in Hsieh et al. (2010)	GQ502699	GQ455443	GQ848364	MZ854248
*X. flabelliformis* (Schwein.) Fr.	French West Indies	*YMJ515* from *Lechat*,* C. CLL5121* (Fournier et al. 2019), as *X*. *cubensis* in Hsieh et al. (2010)	GQ502701	GQ455445	GQ848366	GU373810
*X. frustulosa* (Berk. & M. A. Curtis) Cooke	French West Indies	*YMJ771* from *Lechat*,* C. CLL6002-2* (Fournier et al. 2018; Hsieh et al. 2010)	GQ495943	GQ449237	GQ844837	GU322450
*X. frustulosa* (Berk. & M. A. Curtis) Cooke	Taiwan	*Ju & Hsieh 92092010* (Hsieh et al. 2010)	GQ495944	GQ449240	GQ844838	GU322451
*X. fulvescens* N. Wangsawat et al.	Thailand	HOLOTYPE (Wangsawat et al. 2021)	–	–	–	*MT622780*
*X. furcata* Fr.	Indonesia	NEOTYPE (Ju et al. 2023)	–	–	–	*OQ848122*
*X. furcata* Fr.	Indonesia	Stromata emerging from fungus comb (FH) (Ju et al. 2023)	–	–	–	*OQ848123*
*X. furcata* Fr.	Taiwan	*YMJ646* (Ju et al. 2023), as *X.* sp. 4 in Hsieh et al. (2010)	GQ502715	GQ853050	GQ853032	*GU324757*
*X. furcata* Fr.	Taiwan	*YMJ956* (Ju et al. 2023)	OQ818449	OQ818389	OQ851543	*OQ842973*
*X. furcata* Fr.	Taiwan	*YMJ978* (Ju et al. 2023)	OQ818450	OQ818390	OQ851544	*OQ842974*
*X. furcata* Fr.	Taiwan	*YMJ994* (Ju et al. 2023)	OQ818454	OQ818394	OQ851548	*OQ842978*
*X. furcata* Fr.	Taiwan	*YMJ1102* (Ju et al. 2023)	OQ818457	OQ818397	OQ851551	*OQ842981*
*X. furcata* Fr.	Taiwan	*YMJ1123* (Ju et al. 2023)	OQ818461	OQ818401	OQ851555	*OQ842985*
*X. furcata* Fr.	Taiwan	*YMJ1133* (Ju et al. 2023)	OQ818468	OQ818408	OQ851562	*OQ842992*
*X. furcata* Fr.	Taiwan	*YMJ1730* from EPITYPE (Ju et al. 2023)	OQ818471	OQ818411	OQ851565	*OQ842995*
*X. furcata* Fr.	Taiwan	*YMJ1737* from EPITYPE (Ju et al. 2023)	OQ818475	OQ818415	OQ851569	*OQ842999*
*X. furcata* Fr.	Taiwan	*YMJ1738* from EPITYPE (Ju et al. 2023)	OQ818476	OQ818416	OQ851570	*OQ843000*
*X. furcatula* Y.-M. Ju & H.-M. Hsieh	Taiwan	*YMJ1099* from HOLOTYPE (Ju et al. 2023)	OQ818433	OQ818373	OQ851527	*OQ845483*
*X. furcatula* Y.-M. Ju & H.-M. Hsieh	Taiwan	*YMJ1103* from HOLOTYPE (Ju et al. 2023)	OQ818434	OQ818374	OQ851528	*OQ845484*
*X. furcatula* Y.-M. Ju & H.-M. Hsieh	Taiwan	*YMJ1918* (Ju et al. 2023)	OQ818437	OQ818377	OQ851531	*OQ845487*
*X. furcatula* Y.-M. Ju & H.-M. Hsieh	Taiwan	*YMJ2194* (Ju et al. 2023)	OQ818438	OQ818378	OQ851532	*OQ845488*
*X. globosa* (Spreng. ex Fr.) Mont.	French West Indies	*YMJ775* from *Lechat*,* C. CLL6033* (Hsieh et al. 2010)	GQ502684	GQ452369	GQ848348	GU324735
*X. grammica* (Mont.) Fr.	Taiwan	*YMJ479* from *Chen*,* G.-T.* (Hsieh et al. 2010)	GQ487704	GQ427197	GQ844813	GU300097
*X. griseosepiacea* Y.-M. Ju & H.-M. Hsieh	Taiwan	*YMJ641* from HOLOTYPE (Ju and Hsieh 2007)	GQ502714	GQ853049	GQ853031	*EU179865*
*X. guepini* (Fr.) Fr.	France	*D. Huart*,* HD 20120101* (Hsieh et al. 2022)	OP856892	OP856890	OP856891	*OP863307*
*X. haemorrhoidalis* Berk. & Broome	Taiwan	*Ju & Hsieh 89041207* (Hsieh et al. 2010)	GQ502683	GQ452368	GQ848347	GU322464
*X. hoehnelii* Y.-M. Ju & H.-M. Hsieh	Taiwan	*YMJ642* from HOLOTYPE (Ju et al. 2023), as *X*. sp. 1 in Hsieh et al. (2010)	GQ502719	GQ853054	GQ853036	*GU324759*
*X. hoehnelii* Y.-M. Ju & H.-M. Hsieh	Taiwan	*YMJ643* from HOLOTYPE (Ju et al. 2023)	OQ818439	OQ818379	OQ851533	*OQ845489*
*X. hoehnelii* Y.-M. Ju & H.-M. Hsieh	Taiwan	*YMJ1159* (Ju et al. 2023)	OQ818443	OQ818383	OQ851537	*OQ845493*
*X. hoehnelii* Y.-M. Ju & H.-M. Hsieh	Taiwan	*YMJ2178* (Ju et al. 2023)	OQ818444	OQ818384	OQ851538	*OQ845494*
*X.* cf. *heliscus* (Mont.) J. D. Rogers & Y.-M. Ju	Taiwan	*Ju & Hsieh 88113010* (Hsieh et al. 2010)	GQ502691	GQ452376	GQ848355	GU324742
*X. hypoxylon* (L.) Grev.	Belgium	*YMJ152* from *Ju*,* Y.-M.* (Hsieh et al. 2010)	GQ260187	GQ427196	GQ844812	*GU300096*
*X. hypoxylon* (L.) Grev.	Taiwan	*Guu*,* J.-R. 95082001* (Hsieh et al. 2010)	GQ487703	GQ427195	GQ844811	GU300095
*X. ianthinovelutina* (Mont.) Fr.	French West Indies	*YMJ553* from *Lechat*,* C. CLL5599* (Hsieh et al. 2010)	GQ495934	GQ438755	GQ844828	GU322441
*X. insignifurcata* Y.-M. Ju & H.-M. Hsieh	Taiwan	*YMJ650* from HOLOTYPE (Ju et al. 2023), as *X*. sp. 5 in Hsieh et al. (2010)	GQ502716	GQ853051	GQ853033	*GU324758*
*X. insignifurcata* Y.-M. Ju & H.-M. Hsieh	Taiwan	*YMJ649* from HOLOTYPE (Ju et al. 2023)	OQ818446	OQ818386	OQ851540	*OQ845496*
*X. insignifurcata* Y.-M. Ju & H.-M. Hsieh	Taiwan	*YMJ1198* (Ju et al. 2023)	OQ818447	OQ818387	OQ851541	*OQ845497*
*X. insolita* Y.-M. Ju et al.	Taiwan	*YMJ1251* from HOLOTYPE (Hsieh et al. 2020)	MN656983	MN656985	MN656981	*MN655979*
*X. intracolorata* (J. D. Rogers et al.) J. D. Rogers & Y.-M. Ju	Taiwan	*Ju & Hsieh 90080402* (Hsieh et al. 2010)	GQ502690	GQ452375	GQ848354	GU324741
*X. intraflava* Y.-M. Ju & H.-M. Hsieh	Taiwan	*YMJ725* from HOLOTYPE (Ju and Hsieh 2007)	GQ502718	GQ853053	GQ853035	*EU179866*
*X. iriomotensis* Okane et al.	Japan	HOLOTYPE (the present study)	**PQ605185**	**PQ605195**	**PQ605190**	***AB274815***
*X. ischnostroma* Wangsawat et al.	Thailand	*SWUF18-22.1* from HOLOTYPE (Wangsawat et al. 2021)	OQ845435	OQ845427	OQ851587	*MT622788*
*X. juruensis* Henn.	Taiwan	*Ju & Hsieh 92042501* (Hsieh et al. 2010)	GQ495932	GQ438753	GQ844825	GU322439
*X. liquidambar* J. D. Rogers et al.	Taiwan	*Ju & Hsieh 93090701* (Hsieh et al. 2010)	GQ487702	GQ421290	GQ844810	GU300094
*X. margaretae* N. Wangsawat et al.	Thailand	HOLOTYPE (Wangsawat et al. 2021)	–	–	–	*MT622778*
*X. martinicensis* J. Fourn. & Lechat	French West Indies	*YMJ508* ISOTYPE (HAST145132) (Fournier et al. 2020), also in Hsieh et al. (2010), as *X*. *luteostromata* var. *macrospora*	GQ502688	GQ452373	GQ848352	GU324739
*X. meliacearum* Læssøe	Puerto Rico	*YMJ148* from *Lodge*,* D. J. PR-894* (Læssøe and Lodge 1994)	GQ478219	GQ408906	GQ844797	GU300084
*X. mianyangensis* Y.-M. Ju et al.	China	*YMJ1321* from HOLOTYPE (Ju et al. 2022)	MZ901319	MZ901347	MZ901333	*MZ888986*
*X. microceras* (Mont.) Fr.	French West Indies	*YMJ414* from *Lechat*,* C. CLL2265* (Hsieh et al. 2010)	GQ478221	GQ408908	GQ844799	GU300086
*X. minima* Wangsawat et al.	Thailand	*SWUF18-3.2* from HOLOTYPE (Wangsawat et al. 2021)	OQ845436	OQ845428	OQ851588	*MT622789*
*X. multiplex* (Kunze) Fr.	French West Indies	*YMJ580* from *Lechat*,* C. CLL5287* (Hsieh et al. 2010)	GQ487705	GQ427198	GQ844814	GU300098
*X. multiplex* (Kunze) Fr.	USA, Hawaiian Islands	*YMJ259* from *Hemmes*,* D. E. Xy-7* (Hsieh et al. 2010)	GQ487706	GQ438743	GQ844815	GU300099
*X. muscula* C. G. Lloyd	French West Indies	*YMJ520* from *Lurel*,* D. CLL5323* (Hsieh et al. 2010)	GQ478222	GQ408909	GQ844800	GU300087
*X. neonigripes* Y.-M. Ju et al.	China	*WLS2030* (Ju et al. 2022)	MZ901320	MZ901348	MZ901334	*MZ888987*
*X. neonigripes* Y.-M. Ju et al.	Taiwan	*YMJ722* from *Chou*,* K.-H. 95060503* (Ju et al. 2022), as *X*. sp. 3 in Hsieh et al. (2010)	GQ502712	GQ853047	GQ853029	*GU324756*
*X. neonigripes* Y.-M. Ju et al.	Taiwan	*YMJ1202* from HOLOTYPE (Ju et al. 2022)	MZ901321	MZ901349	MZ901335	*MZ888988*
*X. nigripes* (Klotzsch) Fr.	China	*WLS2056* (Ju et al. 2022)	MZ901322	MZ901350	MZ901336	*MZ888989*
*X. nigripes* (Klotzsch) Fr.	Taiwan	*YMJ653* from *Chou*,* K.-H. 94053001* (Ju and Hsieh 2007; Ju et al. 2022)	GQ502710	GQ853045	GQ853027	*EU179868*
*X. ochraceostroma* Y.-M. Ju & H.-M. Hsieh	Taiwan	*YMJ401* from HOLOTYPE (Ju and Hsieh 2007)	GQ502717	GQ853052	GQ853034	*EU179869*
*X. oligotoma* Sacc. & Paol.	French Guiana	*YMJ784* from *Lechat*,* C. CLL7031* (Hsieh et al. 2010)	GQ487700	GQ421288	GQ844808	GU300092
*X. ophiopoda* Sacc.	Taiwan	*Ju & Hsieh 93082805* (Hsieh et al. 2010)	GQ495955	GQ452365	GQ848344	GU322461
*X. oxyacanthae* Tul. & C. Tul.	USA	*YMJ859* from *Yeomans*,* R.* (Hsieh et al. 2010)	GQ495927	GQ438748	GQ844820	GU322434
*X. palmicola* G. Winter	New Zealand	*YMJ604* from *Samuels*,* G. J. 85−83* (Hsieh et al. 2010)	GQ495929	GQ438750	GQ844822	GU322436
*X. papulis* C. G. Lloyd	Taiwan	*Ju & Hsieh 89021903* (Hsieh et al. 2010)	GQ487707	GQ438744	GQ844816	GU300100
*X. phyllocharis* Mont.	French West Indies	*YMJ528* from *Lechat*,* C. CLL5302* (Hsieh et al. 2010)	GQ495938	GQ449234	GQ844832	GU322445
*X. plebeja* Ces.	Taiwan	*Ju & Hsieh 91122401* (Hsieh et al. 2010)	GQ502689	GQ452374	GQ848353	GU324740
*X. polymorpha* (Pers.) Grev.	USA	*YMJ1012* from *Rogers*,* J. D.* (Hsieh et al. 2010)	GQ495954	GQ452364	GQ848343	GU322460
*X. reevesiae* Y.-M. Ju et al.	Taiwan	From HOLOTYPE (Ju et al. 2018), as *X*. sp. 7 in Hsieh et al. (2010)	GQ495928	GQ438749	GQ844821	GU322435
*X. regalis* Cooke	India	*YMJ920* from *Gailawad*,* S. AMH 9204* (Hsieh et al. 2010)	GQ502694	GQ452379	GQ848358	GU324745
*X. regalis* Cooke	Taiwan	*Ju & Hsieh 92072001* (Hsieh et al. 2010)	GQ502693	GQ452378	GQ848357	GU324744
*X. reinkingii* var. *microspora* N. Wangsawat et al.	Thailand	HOLOTYPE (Wangsawat et al. 2021)	–	–	–	*MT622769*
*X. rhytidosperma* J. Fourn. & Lechat	French West Indies	*YMJ431* from ISOTYPE (Fournier et al. 2018b), as *X*. cf. *glebulosa* in Hsieh et al. (2010)	GQ495956	GQ452366	GQ848345	GU322462
*X. ripicola* C. S. Kim & S.-K. Han	South Korea	HOLOTYPE (Kim et al. 2016)	–	–	–	*KM817199*
*X. rogersionigripes* Y.-M. Ju et al.	China	*YMJ1443* (Ju et al. 2022)	–	–	–	*MZ889001*
*X. rogersionigripes* Y.-M. Ju et al.	China	*YMJ1148* (Ju et al. 2022)	–	–	–	*MZ889000*
*X. rogersionigripes* Y.-M. Ju et al.	China	*WLS2064* (Ju et al. 2022)	MZ901323	MZ901351	MZ901337	*MZ888990*
*X. rogersionigripes* Y.-M. Ju et al.	Taiwan	*YMJ1791* from HOLOTYPE (Ju et al. 2022)	MZ901324	MZ901352	MZ901338	*MZ888991*
*X. rogersionigripes* Y.-M. Ju et al.	Taiwan	*YMJ1795* from HOLOTYPE (Ju et al. 2022)	MZ901325	MZ901353	MZ901339	*MZ888992*
*X. schweinitzii* Berk. & M. A. Curtis	Taiwan	Ju & Hsieh 9209 2023 (Hsieh et al. 2010)	GQ495957	GQ452367	GQ848346	GU322463
*X. scoparia* Pat.	Taiwan	*YMJ960* (Ju et al. 2023)	OQ818478	OQ818418	OQ851572	*OQ843003*
*X. scoparia* Pat.	Taiwan	*YMJ989* (Ju et al. 2023)	OQ818481	OQ818421	OQ851575	*OQ843006*
*X. scoparia* Pat.	Taiwan	*YMJ1158* (Ju et al. 2023)	OQ818484	OQ818424	OQ851578	*OQ843009*
*X. scoparia* Pat.	Taiwan	*YMJ1428* (Ju et al. 2023)	OQ818488	OQ818428	OQ851582	*OQ843013*
*X. scoparia* Pat.	Taiwan	*YMJ1435* from EPITYPE (Ju et al. 2023)	OQ818489	OQ818429	OQ851583	*OQ843014*
*X. scruposa* (Fr.) Fr.	French West Indies	*YMJ497* from *Lechat*,* C. CLL5025* (Hsieh et al. 2010)	GQ495952	GQ452362	GQ848341	GU322458
*X. siamensis* Wangsawat et al.	Thailand	*SWUF17-20.2* from HOLOTYPE (Ju et al. 2023; Wangsawat et al. 2021)	OQ845437	OQ845429	OQ851589	*MT622765*
*X. sihanonthii* Wangsawat et al.	Thailand	*SWUF18-1.3* from HOLOTYPE (Wangsawat et al. 2021)	–	–	–	*MT622785*
*X.* sp. A	China	*WLS2062* (Ju et al. 2022)	MZ901326	MZ901354	MZ901340	*MZ888993*
*X.* sp. A	China	*WLS2069* (Ju et al. 2022)	MZ901327	MZ901355	MZ901341	*MZ888994*
*X.* sp. A	China	*WLS2077* (Ju et al. 2022)	MZ901328	MZ901356	MZ901342	*MZ888995*
*X.* sp. B	China	*WLS2084* (Ju et al. 2022)	MZ901329	MZ901357	MZ901343	*MZ888996*
*X. striata* Pat.	China	*YMJ304* from *Leu*,* L.-S.* (Hsieh et al. 2010)	GQ478224	GQ421284	GQ844803	GU300089
*X. subescharoidea* Y.-M. Ju et al.	China	*YMJ1442* (Ju et al. 2022)	MZ901330	MZ901358	MZ901344	*MZ888997*
*X. subescharoidea* Y.-M. Ju et al.	China	*YMJ1484* (Ju et al. 2022)	MZ901331	MZ901359	MZ901345	*MZ888998*
*X. subescharoidea* Y.-M. Ju et al.	China	*WLS2026* (Ju et al. 2022)	MZ901332	MZ901360	MZ901346	*MZ888999*
*X. subescharoidea* Y.-M. Ju et al.	Taiwan	*YMJ1188* from HOLOTYPE (Hsieh et al. 2020; Ju et al. 2022)	MN656984	MN656986	MN656982	*MN655980*
*X. subescharoidea* Y.-M. Ju et al.	Taiwan	*YMJ660* from *Chou*,* K.-H. 95052301* (Ju et al. 2022), as *X*. sp. 2 in Hsieh et al. (2010); immature	GQ502708	GQ853043	GQ853025	*GU324754*
*X. subintraflava* Wangsawat et al.	Thailand	*SWUF16-4.3* (Wangsawat et al. 2021)	OQ845438	OQ845430	OQ851590	*MT622762*
*X. telfairii* (Berk.) Fr.	French West Indies	*YMJ421* from *Lechat*,* C. CLL2224* (Fournier et al. 2019; Hsieh et al. 2010)	GQ502686	GQ452371	GQ848350	GU324731
*X. telfairii* (Berk.) Fr.	Taiwan	*Ju & Hsieh 90081901* (Hsieh et al. 2010)	GQ502687	GQ452372	GQ848351	GU324738
*X. tenellifurcata* Y.-M. Ju & H.-M. Hsieh	Taiwan	*YMJ1070* from HOLOTYPE (Ju et al. 2023)	OQ818448	OQ818388	OQ851542	*OQ845498*
*X. terricola* Y.-M. Ju et al.	Taiwan	*YMJ1375* from HOLOTYPE (Chou et al. 2017)	MF577044	MF577045	MF577043	*MF577042*
*X. terricola* Y.-M. Ju et al.	Taiwan	*Chou*,* W.-N. CWN08372* (Chou et al. 2017)	MF577040	MF577041	MF577039,	*MF577038*
*X. termiticola* (Okane & Nakagiri) Y.-M. Ju	Japan	HOLOTYPE (Okane and Nakagiri 2007), as *Geniculisynnema termiticola*	**PQ605186**	**PQ605196**	**PQ605191**	*** AB274813***
*X. theinhirunae* Wangsawat et al.	Thailand	*SWUF16-10.1* (Wangsawat et al. 2021)	OQ845439	OQ845431	OQ851591	*MT622773*
*X. theinhirunae* Wangsawat et al.	Thailand	*SWUF17-44.1* from HOLOTYPE (Wangsawat et al. 2021)	OQ845440	OQ845432	OQ851592	*MT622771*
*X. tuberoides* Rehm	French West Indies	*YMJ475* from *Lechat*,* C. CLL2146* (Fournier et al. 2019; Hsieh et al. 2010)	GQ478209	GQ398232	GQ844784	GU300074
*X. vagans* Petch	USA, Hawaiian Islands	*YMJ258* from *Hemmes*,* D. E. DEH-1052*,* as X.* sp. 6 in Hsieh et al. (2010)	GQ478217	GQ408904	GQ844795	GU300082
*X. venosula* Speg.	USA, Hawaiian Islands	*Ju & Hsieh 94080508* (Ju et al. 2007)	EF025617	EF025602	GQ844806	EF026149
*X. venustula* Sacc.	Taiwan	*Ju & Hsieh 88113002* (Hsieh et al. 2010)	GQ487699	GQ421287	GQ844807	GU300091
*X. vinacea* N. Wangsawat et al.	Thailand	HOLOTYPE (Wangsawat et al. 2021)	**PQ605187**	**PQ605197**	**PQ605192**	*MT622783*
*X. vivantii* Y.-M. Ju et al.	French West Indies	*YMJ519* from HOLOTYPE (Ju et al. 2018), as *X*. sp. 8 in Hsieh et al. (2010)	GQ495931	GQ438752	GQ844824	GU322438

**Fig. 1:**
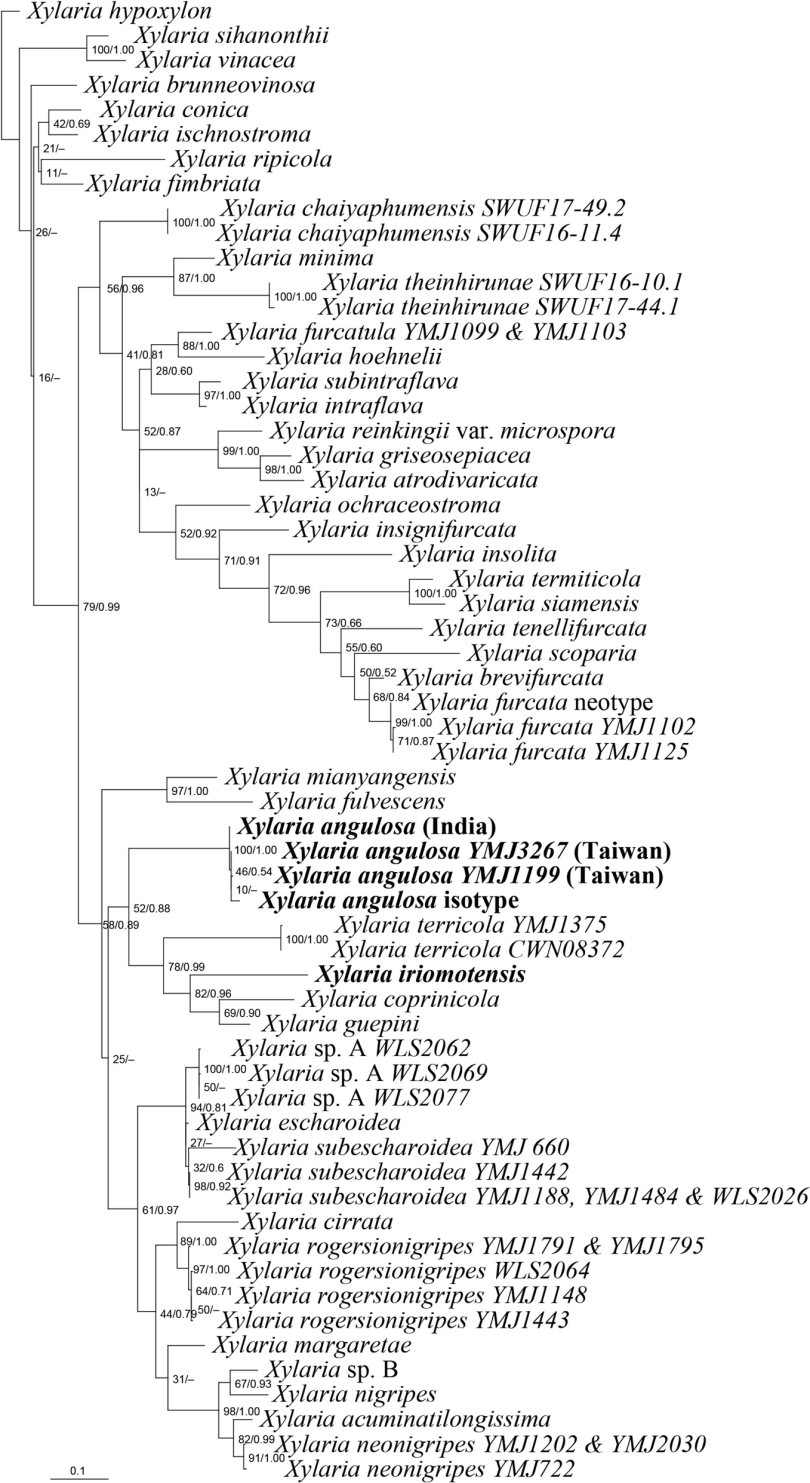
Phylogenetic tree generated by ML analysis from the ITS dataset with the sequence from the WSP isotype of *X*. *angulosa* included. The species with sequences generated in the present study are in boldface. Numbers at internodes represent bootstrap values and are immediately followed by the posterior probability values greater than 50% with BI analysis. *Xylaria hypoxylon* is the outgroup taxon

**Fig. 2:**
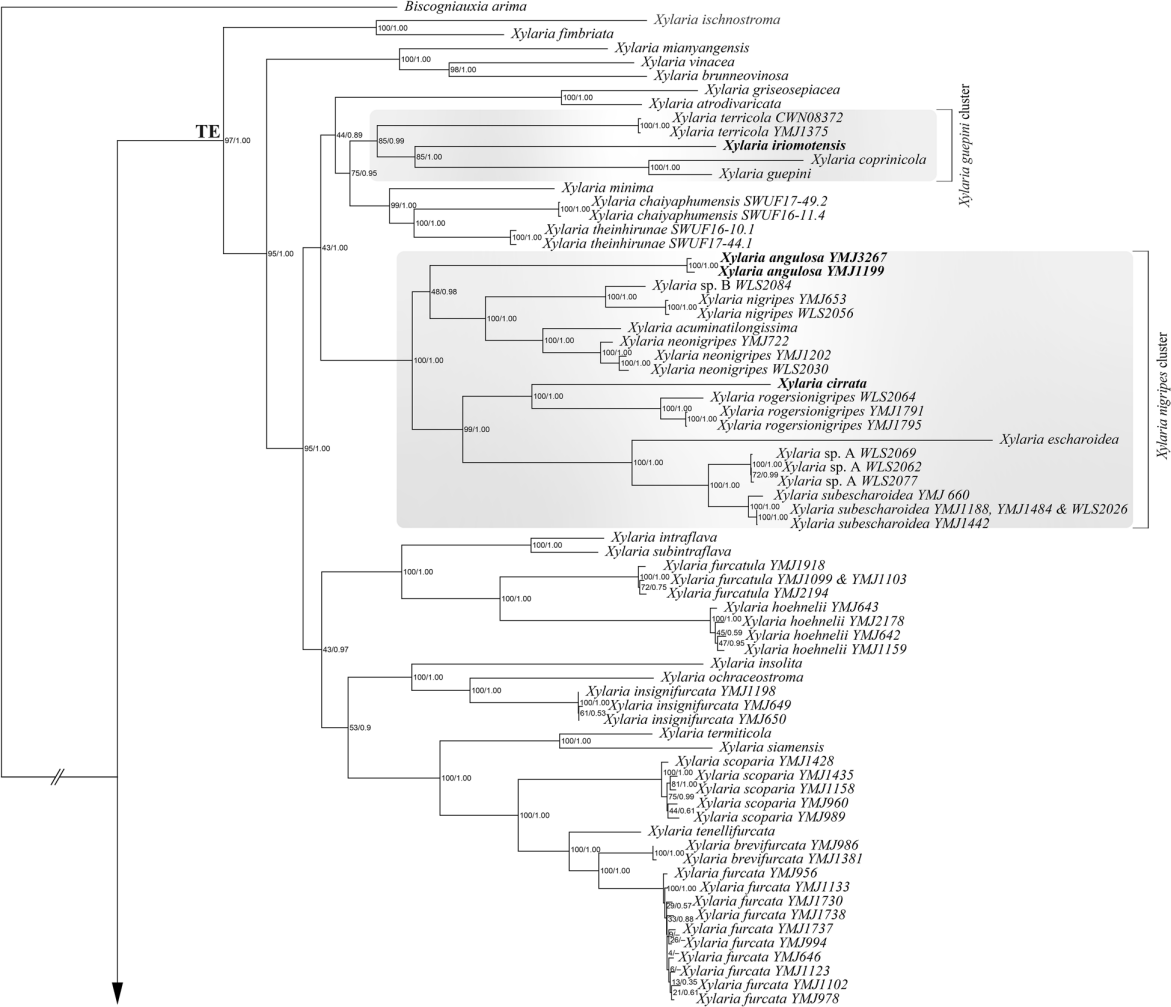

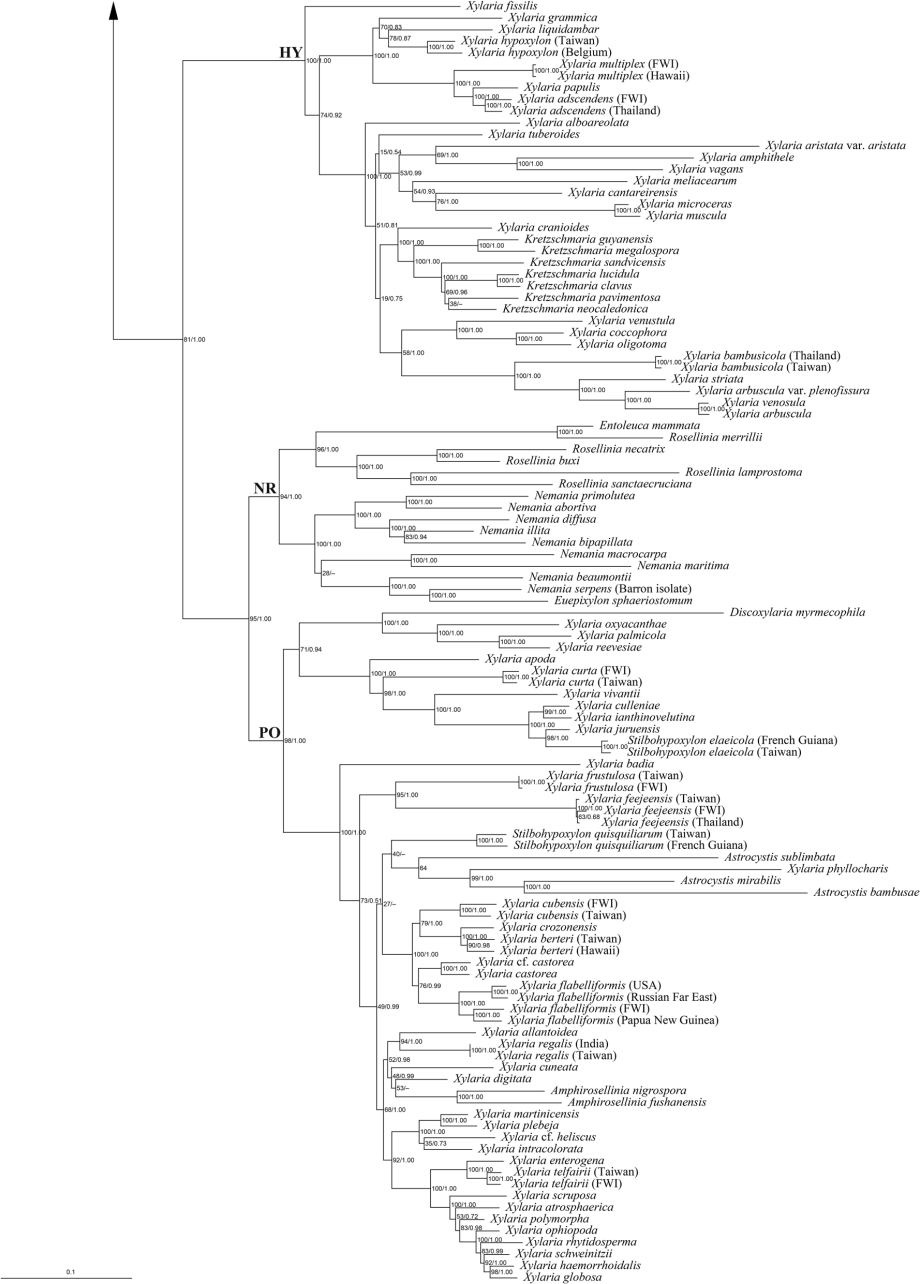
Phylogenetic tree generated by ML analysis from the RPB2-TUB-ACT dataset. The subclades of the TE clade including the *X*. *guepini* cluster and the *X*. *nigripes* cluster are in shade. The species newly described in the present study are in boldface. Numbers at internodes represent bootstrap values and are immediately followed by the posterior probability values greater than 50% with BI analysis. *Biscogniauxia arima* is the outgroup taxon

The 85 italicized ITS sequence accession numbers were analyzed and presented in Fig. 1. Concatenated sequences of β-tubulin gene, α-actin gene, and RPB2 were analyzed and presented in Fig. 2. Sequences in boldface were generated in this study

To calculate the similarities between pairs of ITS sequences from different collections of *X. angulosa*, we used DNADIST from the PHYLIP version 3.6 phylogenetic inference package (Felsenstein 2005).

### Phylogenetic analyses

Sequences of the loci for β-tubulin (*β-TUB*) and α-actin (*α-ACT*) were obtained from the studied *Xylaria* species following Hsieh et al. (2005), while those of the loci for the second largest subunit of RNA polymerase II (*RPB2*) was obtained following Hsieh et al. (2010).

The concatenated sequences of *α-ACT*, *RPB2*, and *β-TUB* from *X*. *angulosa* and *X*. *iriomotensis* were incorporated into the RPB2-TUB-ACT dataset as presented by Ju et al. (2023). This dataset includes the taxa with available sequences listed in Table 1. These concatenated sequences encompass various species of *Xylaria* and its closely related genera. The outgroup used was *Biscogniauxia arima* F. San Martín, Y.-M. Ju & J. D. Rogers. The resulting RPB2-TUB-ACT dataset was aligned using Clustal X 1.81 (Thompson et al. 1997) with “gap penalty” set to 10 and “gap extension penalty” set to 0.2, and was manually improved. It was then subjected to ML and BI analyses. Models of evolution for both ML and BI trees were defined by MrModeltest 2.4 (Nylander 2004), and consensus trees were viewed in FigTree ver. 1.4.4 (http://tree.bio.ed.ac.uk/software/figtree/).

## Results

### Reconfirmation of ***X. angulosa ***and ***X***. ***iriomotensis*** being different species through a comparison of their ITS sequences

The ITS sequences from the isotype of *X*. *angulosa* deposited at WSP and three specimens identifiable as *X*. *angulosa*—two from Taiwan and one from India—exhibited high similarities ranging from 98.28 to 99.66%. These sequences were grouped together using BI and ML phylogenetic methods. This grouping is depicted in Fig. 1, where *X. iriomotensis* was not included in the same group as *X*. *angulosa*. The similarities between the ITS sequences of *X*. *iriomotensis* and *X*. *angulosa* were relatively low, ranging from 84.25 to 85.01%.

### Molecular phylogenetic analyses conducted based on the RPB2‑TUB‑ACT dataset

Both BI and ML analyses based on the RPB2-TUB-ACT dataset were conducted and yielded congruent results. Therefore, only the ML tree is presented (Fig. 2). The results indicate that both *X*. *angulosa* and *X*. *iriomotensis* belong to the TE clade (Hsieh et al. 2010; U’Ren et al. 2016). This clade is equivalent to the subgenus *Pseudoxylaria*, which encompasses *Xylaria* species associated with termite nests and soil. While *X*. *iriomotensis* was grouped within the *X*. *guepini* (Fr.) Fr. cluster with three soil-dwelling species—*X. coprinicola* Y.-M. Ju, H.-M. Hsieh & X.-S. He, *X. guepini*, and *X. terricola* Y.-M. Ju, H.-M. Hsieh & W.-N. Chou, *X*. *angulosa* belonged to the *X*. *nigripes* cluster, which includes all known sclerotium-forming species (Ju et al. 2022). Despite *X*. *angulosa* exhibiting repeatedly dichotomously branched stromata, a characteristic commonly observed in the species of the *X*. *furcata* cluster (Ju et al. 2023), it did not show a close relationship with *X*. *furcata* or its resembling species.

#### Taxonomy

**Fig. 3:**
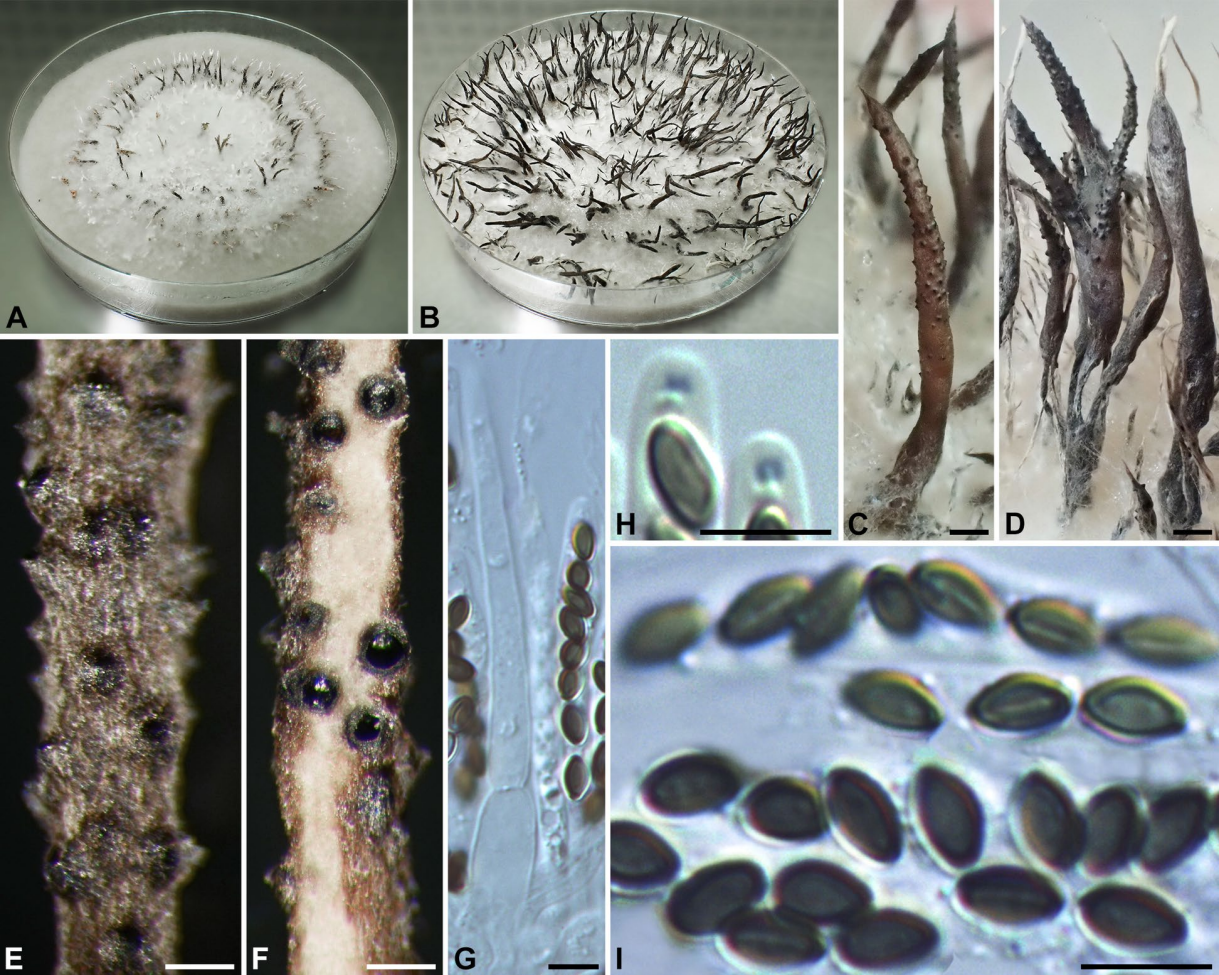
*Xylaria iriomotensis* (from the holotype). **A, B** Colony on 9-cm Petri plate containing OA at 2 and 4 wk, respectively. **C, D** Fresh stromata produced in culture. **E** Surface of a dried stroma showing inconspicuous to conspicuous perithecial mounds and conic-papillate ostioles. **F** Vertical section of a stroma. **G** Asci and a paraphysis. **H** Ascal apical rings and ascospores. **I** Ascospores, with some showing a germ slit. Bars in C, D = 1 mm; E, F = 0.25 mm; G–I = 5 μm

***Xylaria iriomotensis*** I. Okane, H.-M. Hsieh & Y.-M. Ju, sp. nov. Figure 3.

MycoBank MB856549.

**Typification. JAPAN.** Okinawa Pref., Iriomote Island, emerging from incubated termite nests of *Odontotermes formosanus*, 18 Oct 2001, *Okane*,* I.* (cultured), as *X*. *angulosa* (holotype of *X*. *iriomotensis* HAST 146312), culture accession number: NBRC 33288.

**Etymology.** Referring to Iriomote Island where the termite nests were collected.

Colonies on OMA reaching the edge of 9-cm Petri dish in 3 wk, white initially, becoming light grayish brown, appressed to slightly cottony, zonate, with diffuse margins. Reverse uncolored. Stromata arising from concentric zones, cylindrical at fertile part, unbranched or branched occasionally, with an apiculate to acicular apex, on a short stipe or nearly sessile, 0.5–2 cm long in total length × 0.6–1.5 mm diam; surface dark copper brown to dark brown, with inconspicuous to conspicuous perithecial mounds when dried, lacking an outer layer, underlain with a thin, soft layer 20–30 μm thick, concolorous with the surface; interior whitish, homogeneous, soft. Perithecia spherical, 150–250 μm broad. Ostioles conic-papillate, 80–100 μm high × 110–140 μm broad at base. Asci with eight ascospores arranged in uniseriate to partially biseriate manner, cylindrical, 45–55 μm total length, the spore-bearing part 25–45 μm long × 3–4.5 μm broad, with an apical ring staining light blue in Melzer’s iodine reagent, inverted hat-shaped, 1–1.5 μm high × 1–1.5 μm broad. Ascospores brown to dark brown, unicellular, ellipsoid to short fusoid, slightly inequilateral, with narrowly rounded ends, smooth, (3.5–)4.0–4.5(–5.0) × 2.0–2.5(–3.0) µm (4.3 ± 0.3 × 2.4 ± 0.2 μm, *N* = 80), with a straight germ slit slightly less than spore-length to nearly spore-length on the ventral side, lacking a hyaline sheath; epispore smooth. Paraphyses hyaline, tapering upwards, extending much beyond hymenial layer, unbranched.

Anamorph not produced

**Notes. ***Xylaria iriomotensis* is characterized by dark brown, cylindrical stromata, which are topped with an apiculate to acicular apex and lack an outer layer, and the lack of an anamorph. Its teleomorph, initially emerging from fungus combs incubated under laboratory conditions, is readily produced on agar media (Okane and Nakagiri 2007). In our phylogenetic analyses based on the RPB2-TUB-ACT dataset (Fig. 2), *X*. *iriomotensis* was grouped with the *X*. *guepini* cluster, to which three soil-dwelling species—*X. coprinicola*, *X. guepini*, and *X. terricola*—also belong. *Xylaria coprinicola* and *X*. *guepini* are similar to *X*. *iriomotensis* in ascospore morphology, in lacking a stromatal outer layer, and in being capable of producing the teleomorph in culture, but they mainly differ in possessing an anamorph and being soil-dwelling. *Xylaria terricola* is an anamorphic species and thus morphologically incomparable with *X*. *iriomotensis*.

The material of *X. iriomotensis* was previously identified as *X*. *angulosa* by Okane and Nakagiri (2007). Both species have a sterile stromatal apex, a dark brown stromatal surface, and similar ascospore features. However, *X. iriomotensis* has unbranched or occasionally branched stromata with a homogeneous, whitish interior and inconspicuous to conspicuous perithecial mounds on the stromatal surface. In contrast, *X*. *angulosa* has frequently dichotomously branched stromata with a heterogenous interior, which is white at perithecial layer but black at core, and half-exposed perithecial mounds.

**Fig. 4:**
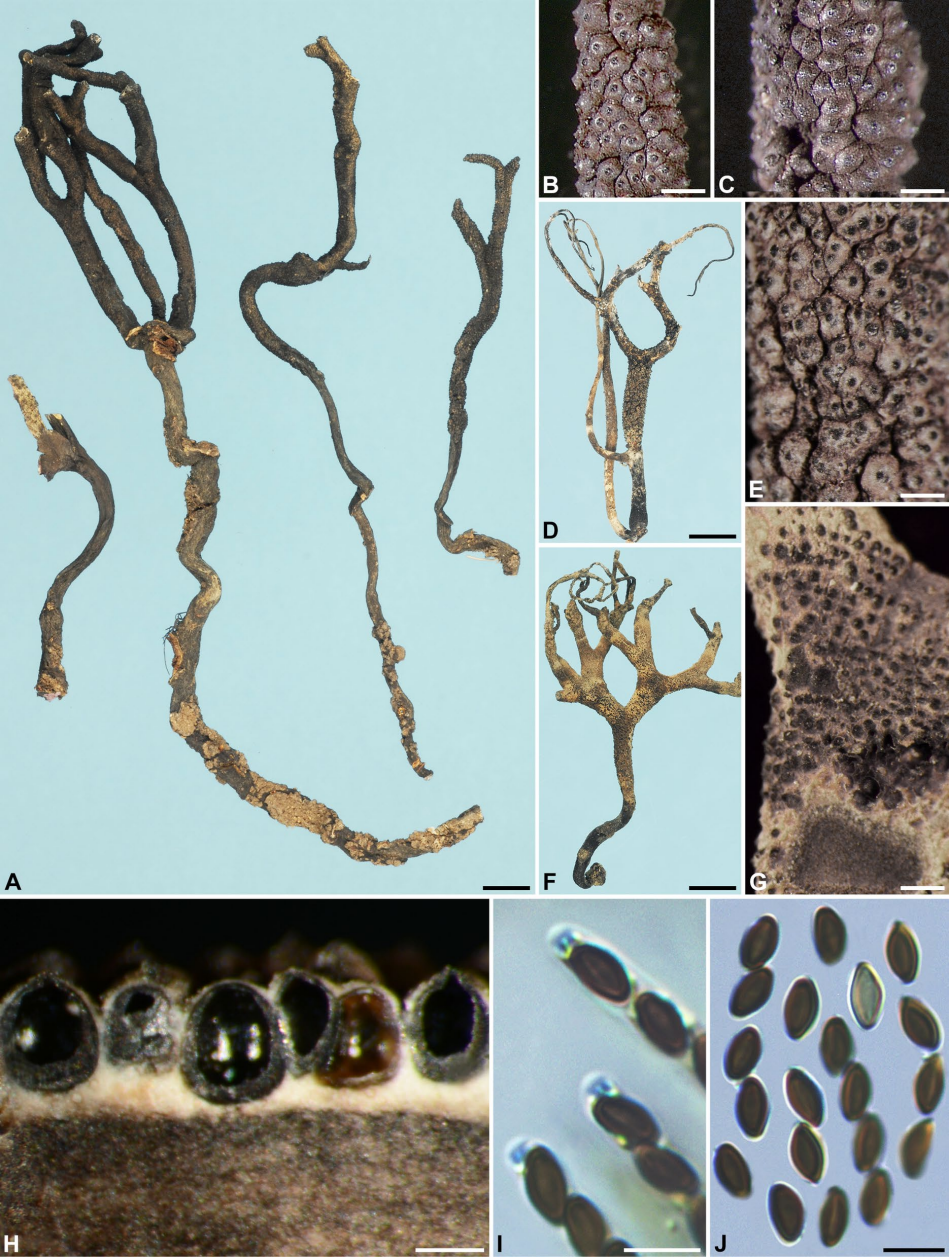
*Xylaria angulosa* (**A–C** from the NY isotype, **D, E, H–J** from HAST 146295, **F, G** from HAST 146296). **A, D, F** Stromata. **B, C, E, G** Stromatal surfaces; the cut in **G** shows the dark core. **H** Vertical section of a stroma. **I** Ascal apical rings and ascospores. **J** Ascospores, with some showing a germ slit. Bars in F = 1 cm; A, D, F = 5 mm; B, C, E, G = 0.5 mm; H = 0.25 mm; I, J = 5 μm

**Fig. 5:**
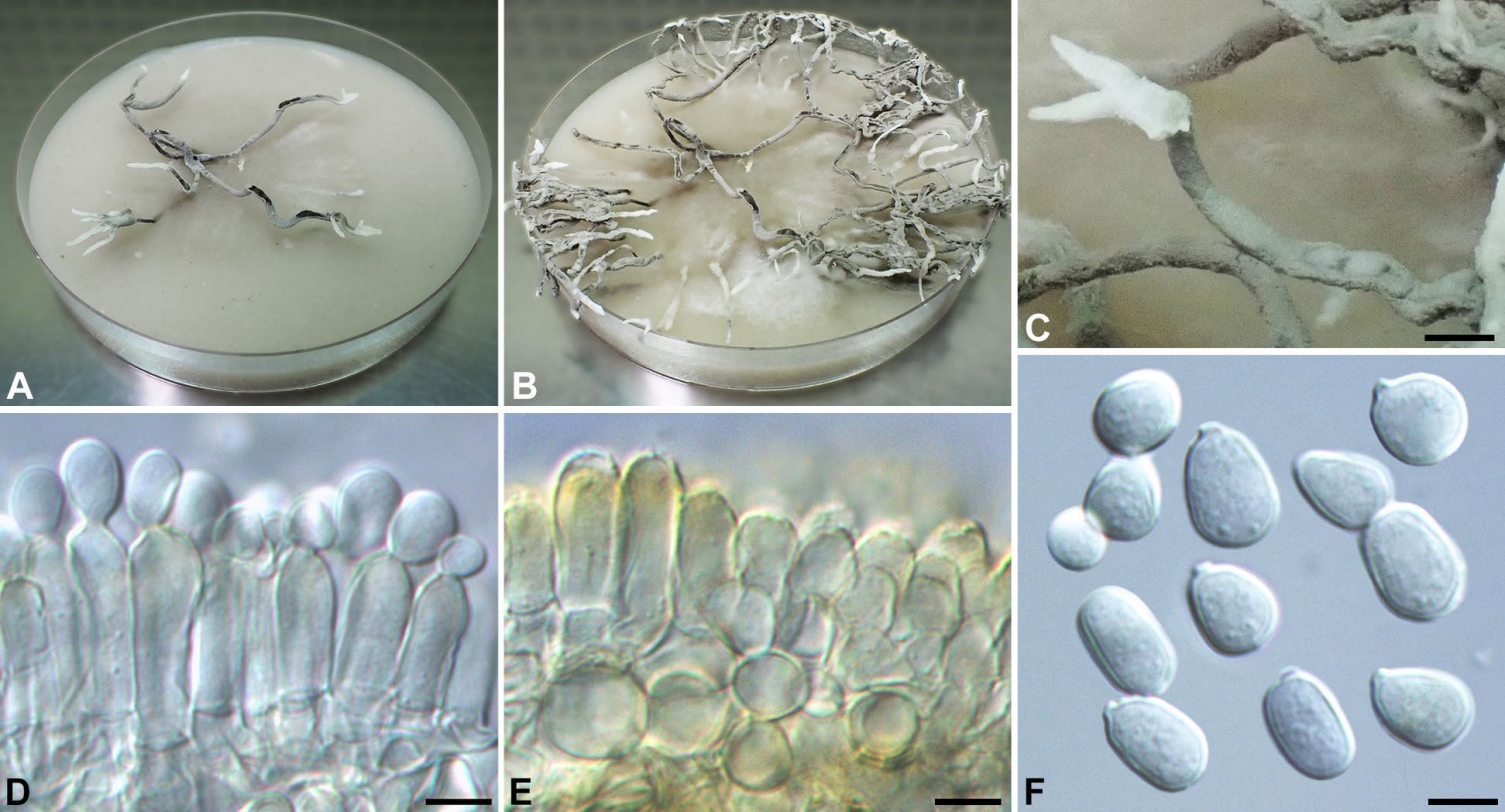
*Xylaria angulosa* (from HAST 146295). **A, B** Colony on 9-cm Petri plate containing OA at 2 and 3 wk, respectively. **C** Stromatal surface overlain with pale mouse gray conidial masses. **D, E** Conidiophores in densely arranged palisades, hyaline initially, becoming yellowish later. **F** Conidia. Bars in C = 2.5 mm; D–F = 5 μm

***Xylaria angulosa*** J. D. Rogers, Callan & Samuels, Mycotaxon 29: 149. 1987. Figures 4 and 5.

Stromata antler-like at fertile part, dichotomously branched one to three times in broad angles or occasionally unbranched or trichotomously branched, with sterile apices short to long, abruptly narrowed when young, broken off when mature, on a glabrous stipe, with a tortuose rooting base, 3.5–5.5 cm long above ground, 1–3 cm long × 2.3–3 mm diam at fertile part; surface dark brown, becoming black, with half-exposed perithecial mounds, lacking an outer layer, underlain with a thin, black layer ca. 10 μm thick; interior white at perithecial layer, with a black core, coriaceous. Perithecia spherical to subspherical, 200–350 μm broad × 200–400 μm high. Ostioles conic-papillate, 50–70 μm high × 90–120 μm broad at base. Asci with eight ascospores arranged in uniseriate manner, cylindrical, 55–75 μm total length, the spore-bearing part 30–40 μm long × 3.5–5 μm broad, with an apical ring staining blue in Melzer’s iodine reagent, inverted hat-shaped, 1–1.5 μm high × 1.5 μm broad. Ascospores brown to dark brown, unicellular, ellipsoid to short fusoid, slightly inequilateral, with narrowly rounded ends, smooth, (4–)4.5–5.5(–6) × (2–)2.5–3(–3.5) µm (5.0 ± 0.3 × 2.6 ± 0.2 μm, *N* = 110), with a straight germ slit spore-length or nearly so on the ventral side, lacking a hyaline sheath; epispore smooth.

Cultures and anamorph. Colonies reaching the edge of 9-cm Petri dish in 3 wk, whitish, mostly submerged, azonate, with diffuse margins. Reverse pale tan-colored. Stromata arising close to the center of the colonies, antler-like, branched several times, up to 6 cm long × 1.2–1.6 mm diam, white, immediately becoming black at base and pale mouse gray on nearly entire surface due to production of conidia. Anamorph produced on the stromatal surface. Conidiophores in upright, densely arranged palisades, dichotomously branched several times from base, smooth, hyaline, becoming yellowish. Conidiogenous cells terminal, cylindrical, 10–15 × 4–5 μm, smooth, bearing one to several terminal poroid conidial secession scars. Conidia produced holoblastically, hyaline, smooth, subglobose to ellipsoid, (6.5–)7–8.5(–10) × (5–)5.5–7.5(–8.5) µm (7.8 ± 0.9 × 6.5 ± 0.9 μm, *N* = 40), with a flattened base indicating former point of attachment to conidiogenous cell.

**Specimens examined**. **INDIA.** Dehra Dun, on termite nests, *Lehmann*,* J.*, as *X. furcata* (WSP ex Rogers herb.). **INDONESIA.** North Sulawesi, Eastern Dumoga-Bone National Park, vic. “Hog’s Back Camp”, on ground, 30–31 Oct 1985, *Samuels*,* G. J. GS2465* (holotype of *X. angulosa* BO 19889, isotypes NY, WSP ex Rogers herb). **TAIWAN.** I-lan Co., Yuan-shan, Fu-shan, on ground, 27 Sep 2014, *Ke*,* Y.-H. 103092701* (cultured from stroma *YMJ3267*) (HAST 146295); Tainan City, Shen-hua District, Liu-fen-liao, on ground, 28 Jun 2010, *Chou*,* K.-H. 99062804* (cultured from stroma *YMJ1199*) (HAST 146296), largely immature.

**Notes.** *Xylaria angulosa* was originally described from North Sulawesi, Indonesia (Rogers et al. 1987). Its stromata are stout and frequently branched dichotomously in broad angles, and have a black core inside, unlike those of *X*. *furcata* and its resembling species (Ju et al. 2023), where stromata are delicate and branched in sharp angles, and lack a black core inside. With the ITS sequence available from the WSP isotype, we reconfirmed that three specimens, two from Taiwan and one from India, are *X. angulosa* despite their stromata being less robust and reduced in size (Fig. 4). The anamorph was observed from the surface of the stromata formed in culture, with conidiophores produced in upright, densely arranged palisades, as opposed to the mononematous conidiophores produced by *X*. *furcata* and its resembling species.

Phylogenetic analyses based on the RPB2-TUB-ACT dataset (Fig. 2) indicated that *X*. *angulosa* grouped within the *X*. *nigripes* cluster, most species of which are capable of forming massive sclerotia (Ju et al. 2022). More field collecting is required to find out if *X*. *angulosa* is another species capable of forming massive sclerotia.

The NY isotype contains two species; besides *X*. *angulosa*, it also contains three stromata of *X*. *brunneovinosa* Y.-M. Ju & H.-M. Hsieh (Hsieh et al. 2020; Ju and Hsieh 2007), which can be distinguished from the former species by its vinaceous-tinged stromatal surface, lack of a dark core inside the stromata, and larger ascospores.

## Discussion

The original material of *X. iriomotensis*, induced from fungus gardens under laboratory conditions in 2001, was reported as *X. angulosa* by Okane and Nakagiri (2007). It remains the only source for *X. iriomotensis*, which is yet to be rediscovered in its natural habitat.

The ITS sequences from the holotype of *X*. *iriomotensis* and the WSP isotype of *X*. *angulosa* allowed us to reconfirm them as two distinct species (Fig. 1). Phylogenetic analyses using sequences of three protein-coding loci, *α-ACT*, *RPB2*, and *β-TUB*, revealed that they are not closely related species. In the phylogenetic tree, *X*. *iriomotensis* is grouped with three soil-dwelling *Xylaria* species of the *X*. *guepini* cluster: *X. coprinicola*, *X. guepini*, and *X. terricola*, while *X*. *angulosa* is placed in the *X*. *nigripes* cluster, which includes species known for producing massive sclerotia within termite nests (Fig. 2).

The grouping of *X*. *iriomotensis*, which lacks an anamorph, with three soil-dwelling species is interesting due to the latter three species possessing highly diversified anamorphs. *Xylaria terricola*, an anamorphic species, produces flabellate stromata resembling those of the *Xylocoremium* state of *X. flabelliformis* (Schwein.) Berk. & M. A. Curtis (Chou et al. 2017). Its conidiophores are loosely arranged, rather than in densely arranged palisades found in most *Xylaria* species. In contrast, *X. coprinicola* and *X*. *guepini* produce mononematous conidiophores, each terminating into an unswollen or swollen top, from which multiple crowded branches arise.

Our phylogenetic analyses suggested that *X*. *angulosa* is a member of the *X*. *nigripes* cluster. The anamorphs of the species in the *X*. *nigripes* cluster are fairly consistent, having their conidiophores in densely arranged palisades, and *X*. *angulosa* is no aberration. The phylogenetic analyses also implied that *X*. *angulosa* may have the potential to form massive sclerotia within termite nests.

## Data Availability

Specimens have been deposited at the HAST herbarium, and cultures are available at BCRC and NBRC. DNA sequences have been deposited at GenBank. Newly described species has been registered at MycoBank.
